# Supplementary material for: Metal-mediated DNA strand displacement and molecular device operations based on base-pair switching of 5-hydroxyuracil nucleobases
Source: Nat Commun. 2023 Aug 24;14:4759. doi: 10.1038/s41467-023-40353-3 (PMC10449808; doi:10.1038/s41467-023-40353-3)
Supplement: Supplementary file 1 — Reporting Summary [file 41467_2023_40353_MOESM1_ESM.pdf]

nature portfolio

Corresponding author(s): Yusuke Takezawa and Mitsuhiko Shionoya

Last updated by author(s): Jun 22, 2023

Reporting Summary

Nature Portfolio wishes to improve the reproducibility of the work that we publish. This form provides structure for consistency and transparency in reporting. For further information on Nature Portfolio policies, see our Editorial Policies and the Editorial Policy Checklist.

Statistics

For all statistical analyses, confirm that the following items are present in the figure legend, table legend, main text, or Methods section.

n/a	Confirmed	
☐	☒	The exact sample size (n) for each experimental group/condition, given as a discrete number and unit of measurement
☒	☐	A statement on whether measurements were taken from distinct samples or whether the same sample was measured repeatedly
☒	☐	The statistical test(s) used AND whether they are one- or two-sided<br>*Only common tests should be described solely by name; describe more complex techniques in the Methods section.*
☒	☐	A description of all covariates tested
☒	☐	A description of any assumptions or corrections, such as tests of normality and adjustment for multiple comparisons
☐	☒	A full description of the statistical parameters including central tendency (e.g. means) or other basic estimates (e.g. regression coefficient) AND variation (e.g. standard deviation) or associated estimates of uncertainty (e.g. confidence intervals)
☒	☐	For null hypothesis testing, the test statistic (e.g. F, t, r) with confidence intervals, effect sizes, degrees of freedom and P value noted<br>*Give P values as exact values whenever suitable.*
☒	☐	For Bayesian analysis, information on the choice of priors and Markov chain Monte Carlo settings
☒	☐	For hierarchical and complex designs, identification of the appropriate level for tests and full reporting of outcomes
☒	☐	Estimates of effect sizes (e.g. Cohen's d, Pearson's r), indicating how they were calculated

Our web collection on statistics for biologists contains articles on many of the points above.

Software and code

Policy information about availability of computer code

Data collection	Gels were imaged on Gel Doc EZ with Image Lab software ver. 6.0.1 (Bio-Rad).
Data analysis	Image Lab ver. 6.1.0 (Bio-Rad) was used for analyzing gel images. All graphs were produced by using KaleidaGraph ver. 5.0.3 (HULINKS). Melting curves were analyzed by using LabSolutions Tm Analysis software ver. 1.40 (Shimadzu).

For manuscripts utilizing custom algorithms or software that are central to the research but not yet described in published literature, software must be made available to editors and reviewers. We strongly encourage code deposition in a community repository (e.g. GitHub). See the Nature Portfolio guidelines for submitting code & software for further information.

Data

Policy information about availability of data

All manuscripts must include a data availability statement. This statement should provide the following information, where applicable:

- Accession codes, unique identifiers, or web links for publicly available datasets
- A description of any restrictions on data availability
- For clinical datasets or third party data, please ensure that the statement adheres to our policy

The experimental data and protocols are provided in the manuscript and the Supplementary Information. Numeric data underlying all plots are available as Source Data, which are provided with this paper.

Research involving human participants, their data, or biological material

Policy information about studies with human participants or human data. See also policy information about sex, gender (identity/presentation), and sexual orientation and race, ethnicity and racism.

Reporting on sex and gender	This research does not involve human participants, their data, or biological material.
Reporting on race, ethnicity, or other socially relevant groupings	This research does not involve human participants, their data, or biological material.
Population characteristics	This research does not involve human participants, their data, or biological material.
Recruitment	This research does not involve human participants, their data, or biological material.
Ethics oversight	This research does not involve human participants, their data, or biological material.

Note that full information on the approval of the study protocol must also be provided in the manuscript.

Field-specific reporting

Please select the one below that is the best fit for your research. If you are not sure, read the appropriate sections before making your selection.

[x] Life sciences [ ] Behavioural & social sciences [ ] Ecological, evolutionary & environmental sciences

For a reference copy of the document with all sections, see nature.com/documents/nr-reporting-summary-flat.pdf

Life sciences study design

All studies must disclose on these points even when the disclosure is negative.

Sample size	No statistical calculation was performed to determine the sample size prior to the experiments. Sample sizes were chosen to show data reproducibility. We typically use at least three replicates.
Data exclusions	No data was excluded from the analysis.
Replication	All experiments were replicated at least three times to verify the reproducibility, and the data are presented as mean ± s.e.m.. We did not find any case where the data was not able to be replicated.
Randomization	Randomization was not relevant to this study because we are not dealing with biological samples/organisms/participants.
Blinding	Blinding was not relevant to this work because we are not dealing with biological samples/organisms/participants.

Reporting for specific materials, systems and methods

We require information from authors about some types of materials, experimental systems and methods used in many studies. Here, indicate whether each material, system or method listed is relevant to your study. If you are not sure if a list item applies to your research, read the appropriate section before selecting a response.

Materials & experimental systems

n/a	Involved in the study	
[x]	[ ]	Antibodies
[x]	[ ]	Eukaryotic cell lines
[x]	[ ]	Palaeontology and archaeology
[x]	[ ]	Animals and other organisms
[x]	[ ]	Clinical data
[x]	[ ]	Dual use research of concern
[x]	[ ]	Plants

Methods

n/a	Involved in the study	
[x]	[ ]	ChIP-seq
[x]	[ ]	Flow cytometry
[x]	[ ]	MRI-based neuroimaging
