# Supplementary material for: Metal-mediated DNA strand displacement and molecular device operations based on base-pair switching of 5-hydroxyuracil nucleobases
Source: Nat Commun. 2023 Aug 24;14:4759. doi: 10.1038/s41467-023-40353-3 (PMC10449808; doi:10.1038/s41467-023-40353-3)
Supplement: Supplementary file 3 — Supplementary information [file 41467_2023_40353_MOESM3_ESM.pdf]

*Supplementary Information*

# Metal-mediated DNA strand displacement and molecular device operations based on base-pair switching of 5-hydroxyuracil nucleobases

Yusuke Takezawa[1]*, Keita Mori[1], Wei-En Huang[1], Kotaro Nishiyama[1], Tong Xing[1], Takahiro Nakama[1] & Mitsuhiko Shionoya[1]*

[1]*Department of Chemistry, Graduate School of Science, The University of Tokyo, 7-3-1 Hongo, Bunkyo-ku, Tokyo 113-0033, Japan*

**Supplementary Table 1.** Sequences of $\mathbf{U^{OH}}$-containing DNA strands used in this study.

| Name | Sequence (5′ to 3′) |
|---|---|
| ODN1 | CAC ATT GTT GTA $\mathbf{U^{OH}U^{OH}U^{OH}}$ $\mathbf{U^{OH}}$ |
| ODN2 | $\mathbf{U^{OH}U^{OH}U^{OH}}$ $\mathbf{U^{OH}}$TA CAA CAA TGT G |
| ODN3 | **AAA A**TA CAA CAA TGT G |
| ODN1′ | CAC ATT GTT GTA $\mathbf{U^{OH}U^{OH}U^{OH}}$ |
| ODN2′ | $\mathbf{U^{OH}U^{OH}U^{OH}}$ TAC AAC AAT GTG |
| ODN3′ | **AAA** TAC AAC AAT GTG |
| ODN4 | **FAM** – AGC TAC CTC CAC ATT GTT GTA $\mathbf{U^{OH}U^{OH}U^{OH}}$ $\mathbf{U^{OH}}$ |
| ODN5 | $\mathbf{U^{OH}U^{OH}U^{OH}}$ $\mathbf{U^{OH}}$TA CAA CAA TGT GGA GGT AGC T – **Dabcyl** |
| ODN6 | **AAA A**TA CAA CAA TGT GGA GGT AGC T |
| ODN7 | **FAM** – AGC TAC CTC CAC ATT GTT GTA $\mathbf{U^{OH}U^{OH}U^{OH}}$ $\mathbf{U^{OH}}$C |
| ODN8 | G$\mathbf{U^{OH}U^{OH}}$ $\mathbf{U^{OH}U^{OH}}$T ACA ACA ATG TGG AGG TAG CT – **Dabcyl** |
| ODN9 | G**AA AA**T ACA ACA ATG TGG AGG TAG CT (– **Dabcyl**) |
| ODN7′ | CAC ATT GTT GTA $\mathbf{U^{OH}U^{OH}U^{OH}}$ $\mathbf{U^{OH}}$C |
| ODN8′ | G$\mathbf{U^{OH}U^{OH}}$ $\mathbf{U^{OH}U^{OH}}$T ACA ACA ATG TG |
| ODN9′ | G**AA AA**T ACA ACA ATG TG |
| ODN7t | **FAM** – AGC TAC CTC CAC ATT GTT GTA **TTT T**C |
| ODN8t | G**TT TT**T ACA ACA ATG TGG AGG TAG CT – **Dabcyl** |
| ODN8s | TAC AAC AAT GTG GAG GTA GCT |
| Tweezer-a | CAA AAC GTG AGA CAC TGG ATC CGA AGC ATT CCA GGT |
| Tweezer-b | **FAM** – TGC CTT GTA AGA GCG ACC ATC AAC CTG GAA TGC TTC GGA T (– **Dabcyl**) |
| Tweezer-c | GGT CGC TCT TAC AAG GCA CAT GAC TAA GAC CAA AAC |
| Tweezer-d | G$\mathbf{U^{OH}U^{OH}}$ $\mathbf{U^{OH}U^{OH}}$G GTG ATA GTC ATG CCA GTG TCA GAC G$\mathbf{U^{OH}U^{OH}}$ $\mathbf{U^{OH}U^{OH}}$G |
| Tweezer-d with T bases | G**TT TT**G GTG ATA GTC ATG CCA GTG TCA GAC G**TT TT**G |
| $U^{OH}$-DNAzyme | GCG GTA CCA GGT CAA AGG TGG GTG AGC C$\mathbf{U^{OH}U^{OH}}$ $\mathbf{U^{OH}}$GA CAA GA$\mathbf{U^{OH}}$ $\mathbf{U^{OH}U^{OH}}$G GCG CGG TTA GAT AGA G |
| T-DNAzyme | GCG GTA CCA GGT CAA AGG TGG GTG AGC C**TT T**GA CAA GA**T TT**G GCG CGG TTA GAT AGA G |
| NaA43 DNAzyme | GCG GCG GTA CCA GGT CAA AGG TGG GTG AGG GGA CGC CAA GAG TCC CCG CGG TTA GAT AGA G |
| substrate | **FAM** – CTC TAT CTA T**rA**G GAA GTA CCG CCG C |

**Supplementary Table 2.** Characterization of $\mathbf{U^{OH}}$-containing strands.

| DNA strands | Composition | Methods | Detected | Calc. | Obs. |
|---|---|---|---|---|---|
| ODN1 | $C_{154}H_{194}N_{49}O_{104}P_{15}$ | ESI | $[M–6H]^{6–}$ | 808.8 | 808.9 |
| ODN2 | $C_{154}H_{192}N_{55}O_{100}P_{15}$ | ESI | $[M–6H]^{6–}$ | 811.8 | 811.8 |
| ODN1′ | $C_{145}H_{183}N_{47}O_{96}P_{14}$ | ESI | $[M–7H]^{7–}$ | 649.4 | 649.4 |
| ODN2′ | $C_{145}H_{181}N_{53}O_{92}P_{14}$ | ESI | $[M–7H]^{7–}$ | 652.1 | 652.1 |
| ODN4 | $C_{267}H_{329}N_{81}O_{167}P_{25}$ | MALDI | $[M–H]^{–}$ | 8117.3 | 8116.6 |
| ODN5 | $C_{264}H_{330}N_{96}O_{160}P_{25}$ | MALDI | $[M–H]^{–}$ | 8180.4 | 8179.2 |
| ODN7 | $C_{276}H_{340}N_{84}O_{173}P_{26}$ | MALDI | $[M–H]^{–}$ | 8400.3 | 8401.3 |
| ODN8 | $C_{274}H_{341}N_{101}O_{166}P_{26}$ | MALDI | $[M–H]^{–}$ | 8505.5 | 8505.2 |
| ODN7′ | $C_{163}H_{206}N_{52}O_{110}P_{16}$ | ESI | $[M–5H]^{5–}$ | 1028.8 | 1028.8 |
| ODN8′ | $C_{164}H_{204}N_{60}O_{106}P_{16}$ | ESI | $[M–4H]^{4–}$ | 1300.7 | 1300.9 |
| Tweezer-d | $C_{347}H_{432}N_{128}O_{230}P_{35}$ | MALDI | $[M–H]^{–}$ | 11158.0 | 11158.1 |
| $U^{OH}$-DNAzyme | $C_{565}H_{697}N_{233}O_{351}P_{57}$ | MALDI | $[M–2H]^{2–}$ | 9065.8 | 9066.6 |

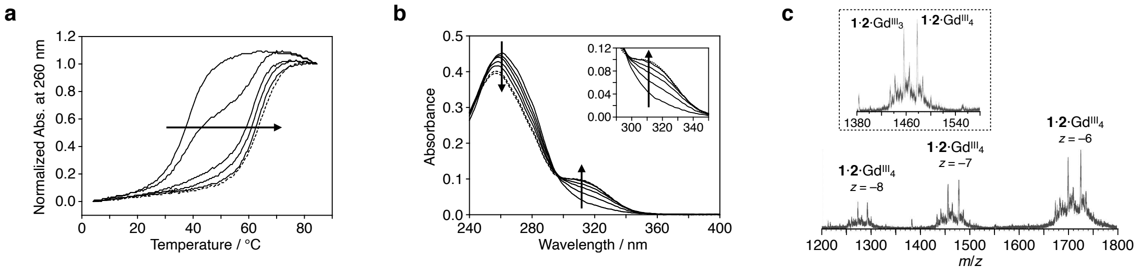


**Supplementary Fig. 1 | $\mathbf{Gd^{III}}$-mediated stabilization of DNA duplex containing $\mathbf{U^{OH}}$–$\mathbf{U^{OH}}$ base pairs.** **a** Melting curves of a DNA duplex containing four $\mathbf{U^{OH}}$–$\mathbf{U^{OH}}$ base pairs (**1**·**2**) in the presence of different concentrations of $Gd^{III}$ ions. 0.2 °C/min. $[Gd^{III}]$/[duplex] = 0, 1, 2, 3, 4 (solid lines), and 6 (broken line). **b** UV absorption spectra of duplex **1**·**2** in the presence of different concentrations of $Gd^{III}$ ions. $[Gd^{III}]$/[duplex] = 0, 1, 2, 3, 4 (solid lines), 5, and 6 (broken lines). In 10 mM HEPES buffer (pH 8.0), 100 mM NaCl. 5 °C, $l$ = 1 cm. The spectra varied almost linearly in the range $[Gd^{III}]$/[duplex] = 0 to 4 and remained almost unchanged in the presence of excess $Gd^{III}$ ions, suggesting that four $Gd^{III}$ ions bind to the four $\mathbf{U^{OH}}$–$\mathbf{U^{OH}}$ base pairs. **c** ESI-TOF mass spectrum of duplex **1**·**2** in the presence of $Gd^{III}$ ions (4 equiv). [duplex] = 40 μM, $[Gd^{III}]$/[duplex] = 4.0 in 20 mM $NH_4OAc$ buffer (pH 7.0). Negative mode. The existence of the trinuclear complex (**1**·**2**·$Gd^{III}_3$) is likely due to the dissociation of the outermost $Gd^{III}$ ion during the measurement. Source data are provided as a Source Data file.

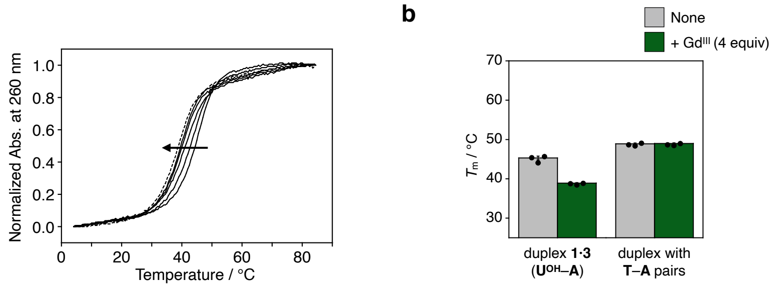


**Supplementary Fig. 2 | Gd$^{III}$-mediated destabilization of DNA duplex containing U$^{OH}$–A base pairs.** **a** Melting curves of a DNA duplex containing four $\mathbf{U^{OH}}$–A base pairs (**1**·**3**) in the presence of different concentrations of Gd$^{III}$ ions. [Gd$^{III}$]/[duplex] = 0, 1, 2, 3, 4 (solid lines), and 6 (broken line) in 10 mM HEPES buffer (pH 8.0), 100 mM NaCl. 0.2 °C/min. **b** Melting temperatures ($T_m$) of duplex **1**·**3** (with $\mathbf{U^{OH}}$–A base pairs) in the absence and presence of Gd$^{III}$ ions (4 equiv). Control experiments with a duplex containing T–A base pairs in place of $\mathbf{U^{OH}}$–A suggested that the Gd$^{III}$-mediated destabilization results from the binding of Gd$^{III}$ ions to the $\mathbf{U^{OH}}$ bases. $N$ = 3 independent experiments. Data are presented as mean values ± SEM. Source data are provided as a Source Data file.

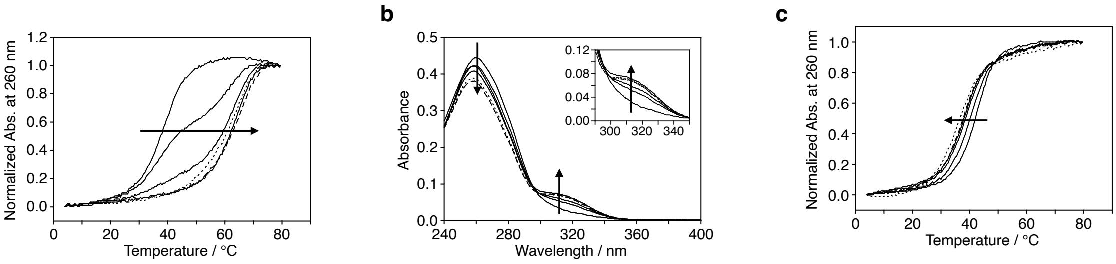


**Supplementary Fig. 3 | Gd$^{III}$-mediated stabilization and destabilization of DNA duplexes containing three U$^{OH}$–U$^{OH}$ or U$^{OH}$–A base pairs.** **a** Melting curves of a DNA duplex containing three $\mathbf{U^{OH}}$–$\mathbf{U^{OH}}$ base pairs (**1′**·**2′**) in the presence of different concentrations of Gd$^{III}$ ions. 0.2 °C/min. **b** UV absorption spectra of duplex **1′**·**2′** in the presence of different concentrations of Gd$^{III}$ ions. 5 °C, $l$ = 1 cm. The spectra varied almost linearly in the range [Gd$^{III}$]/[duplex] = 0 to 3 and remained almost unchanged in the presence of excess Gd$^{III}$ ions, suggesting that three Gd$^{III}$ ions bind to the three $\mathbf{U^{OH}}$–$\mathbf{U^{OH}}$ base pairs. **c** Melting curves of a DNA duplex containing three $\mathbf{U^{OH}}$–A base pairs (**1′**·**3′**) in the presence of different concentrations of Gd$^{III}$ ions. [Gd$^{III}$]/[duplex] = 0, 1, 2, 3 (solid lines), 4 (broken line), and 6 (dotted line), in 10 mM HEPES buffer (pH 8.0), 100 mM NaCl. Source data are provided as a Source Data file.

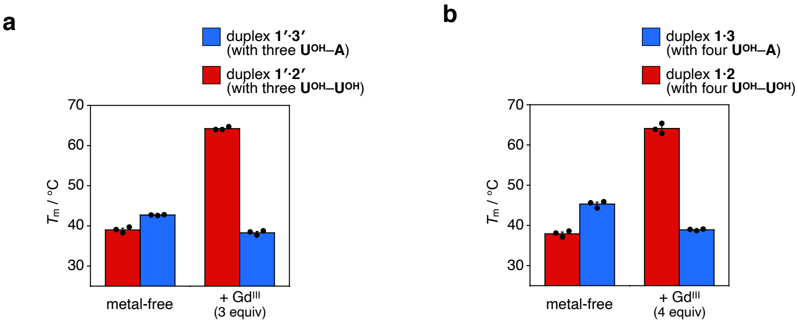


**Supplementary Fig. 4 | Melting temperatures ($T_m$) of duplexes with U$^{OH}$–U$^{OH}$ base pairs and duplexes with U$^{OH}$–A base pairs in the absence and presence of Gd$^{III}$ ions.** **a** Duplex **1′**·**2′** (with three $\mathbf{U^{OH}}$–$\mathbf{U^{OH}}$ pairs) and duplex **1′**·**3′** (with three $\mathbf{U^{OH}}$–**A** pairs). $N$ = 3 independent experiments. Data are presented as mean values ± SEM. **b** Duplex **1**·**2** (with four $\mathbf{U^{OH}}$–$\mathbf{U^{OH}}$ pairs) and duplex **1**·**3** (with four $\mathbf{U^{OH}}$–**A** pairs) (reprinted from Fig. 2b). $N$ = 3 independent experiments. Data are presented as mean values ± SEM. [DNA strand] = 2.0 µM each, [$GdCl_3$] = 0, 6.0, or 8.0 µM (1 equiv for $\mathbf{U^{OH}}$–$\mathbf{U^{OH}}$ pair) in 10 mM HEPES (pH 8.0), 100 mM NaCl. Source data are provided as a Source Data file.

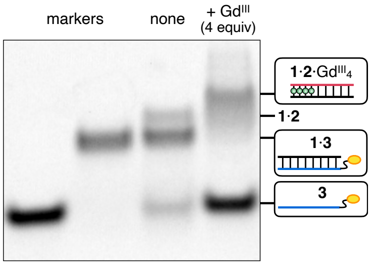


**Supplementary Fig. 5 | Native PAGE analysis of an equimolar mixture of strands 1, 2, and 3 in the absence and presence of Gd$^{III}$ ions (4 equiv).** [DNA duplex] = 2.0 µM, [$GdCl_3$] = 0 or 8.0 µM (1 equiv per **U$^{OH}$–U$^{OH}$** base pair) in 10 mM HEPES (pH 8.0), 100 mM NaCl. The sample was annealed prior to the analysis. Detected after SYBR Gold staining. The authentic samples (strand **3** and pre-annealed duplex **1**·**3**) were employed as the markers. Three independent experiments were performed. Source data are provided as a Source Data file.

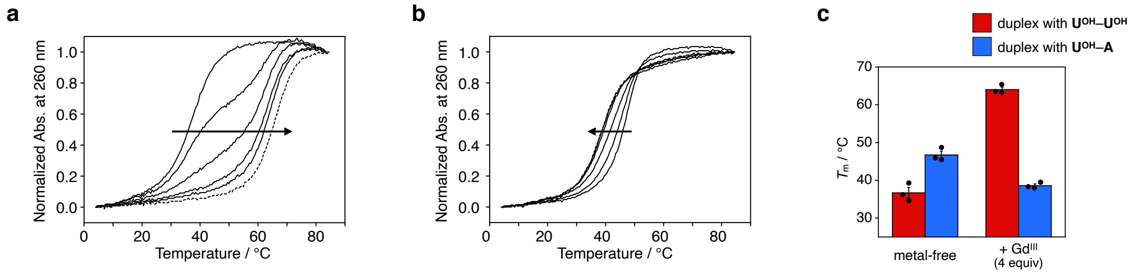


**Supplementary Fig. 6 | Thermal stability of model DNA duplexes containing four U$^{OH}$–U$^{OH}$ or U$^{OH}$–A base pairs with a terminal G–C base pair. a** Melting curves of a duplex containing four **U$^{OH}$–U$^{OH}$** base pairs (**7′**·**8′**) in the presence of different concentrations of Gd$^{III}$ ions. **b** Melting curves of a duplex containing four **U$^{OH}$**–A base pairs (**7′**·**9′**) in the presence of different concentrations of Gd$^{III}$ ions. [Gd$^{III}$]/[duplex] = 0, 1, 2, 3, 4 (solid lines), and 6 (broken line) in 10 mM HEPES buffer (pH 8.0), 100 mM NaCl. 0.2 °C/min. **c** Melting temperatures ($T_m$) of duplexes **7′**·**8′** (with **U$^{OH}$–U$^{OH}$** base pairs) and **7′**·**9′** (with **U$^{OH}$**–A base pairs) in the absence and presence of Gd$^{III}$ ions (4 equiv). $N$ = 3 independent experiments. Data are presented as mean values ± SEM. Shorter DNA strands **7′**, **8′**, and **9′** (17-nt) were utilized because oligonucleotides **7**, **8**, and **9** (26-nt) used in the strand displacement reactions were too long for the melting analysis. **7′**: 5′-CAC ATT GTT GTA **U$^{OH}$U$^{OH}$U$^{OH}$ U$^{OH}$**C-3′, **8′**: 5′-G**U$^{OH}$U$^{OH}$ U$^{OH}$U$^{OH}$**T ACA ACA ATG TG-3′, **9′**: 5′- GAA AAT ACA ACA ATG TG -3′. Source data are provided as a Source Data file.

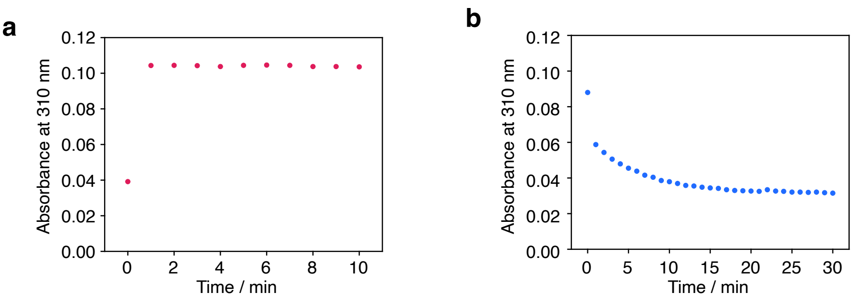


**Supplementary Fig. 7 | Kinetics of metal complexation of DNA duplex 1·2 containing four U$^{OH}$–U$^{OH}$ base pairs. a** Complexation with 4 equiv of Gd$^{III}$ ions. **b** Removal of Gd$^{III}$ ions by equimolar EDTA. [duplex **1**·**2**] = 2.0 µM, [$GdCl_3$] = 8.0 µM (1 equiv per **U$^{OH}$–U$^{OH}$** pair), [EDTA] = 8.0 µM (when applicable) in 10 mM HEPES (pH 8.0), 100 mM NaCl. 25 °C, $l$ = 1 cm. Absorbance at 310 nm, indicative of the **U$^{OH}$**–Gd$^{III}$–**U$^{OH}$** complexes, is plotted. Source data are provided as a Source Data file.

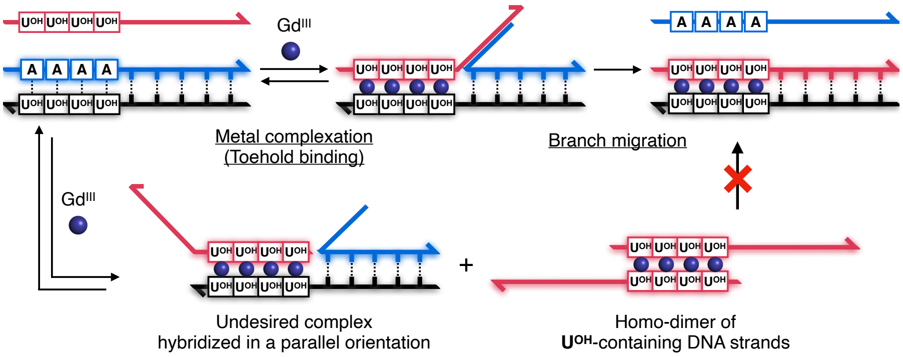


**Supplementary Fig. 8 | A plausible mechanism of the Gd$^{III}$-triggered strand displacement reactions (SDRs).**

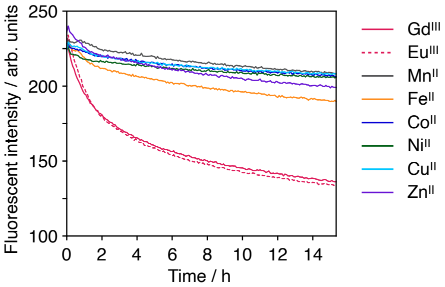


**Supplementary Fig. 9 | Time course analysis of the SDRs triggered by adding various metal ions.** [DNA strand] = 2.0 µM each in 10 mM HEPES buffer (pH 8.0), 100 mM NaCl, 25 °C. The SDRs were monitored with a plate reader by the changes in the fluorescence of FAM ($\lambda_{ex}$ = 495 nm, $\lambda_{em}$ = 520 nm). The invader strand **5** was added to the pre-annealed duplex **4**·**6**, and then the metal ions (4 equiv.) were added to initiate the reaction. Source data are provided as a Source Data file.

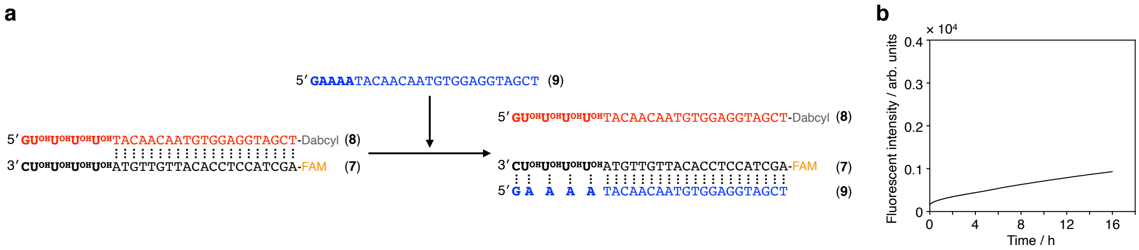


**Supplementary Fig. 10 | Strand displacement reactions (SDRs) using strands 7–9 in the absence of Gd$^{III}$ ions. a** Schematic representation. **b** Time course of the SDRs initiated by the addition of strand **9**. [DNA strand] = 2.0 µM each in 10 mM HEPES buffer (pH 8.0), 100 mM NaCl, 25 °C. The SDRs were monitored by the changes in the fluorescence of the FAM. $\lambda_{ex}$ = 495 nm, $\lambda_{em}$ = 519 nm. Source data are provided as a Source Data file.

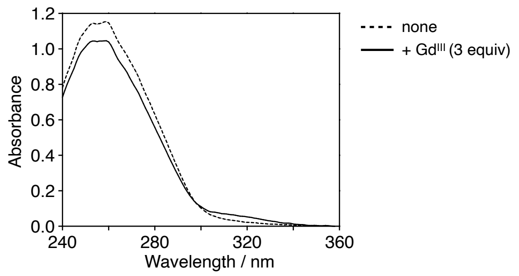


**Supplementary Fig. 11 | UV absorption spectra of $U^{OH}$-DNAzyme in the absence and presence of $Gd^{III}$ ions.** [$\mathbf{U^{OH}}$-DNAzyme] = 20 μM, [$GdCl_3$] = 0 or 60 μM (3 equiv) in 10 mM HEPES (pH 7.0), 100 mM NaCl, $l$ = 0.1 cm, rt. The samples were annealed before the measurement. From the increase in absorbance at 310 nm ($\Delta\varepsilon_{310} = 1.5 \times 10^4$ L·mol$^{-1}$·cm$^{-1}$), it was roughly estimated that more than 70% of the $\mathbf{U^{OH}}$ bases formed the $\mathbf{U^{OH}}$–$Gd^{III}$–$\mathbf{U^{OH}}$ base pairs ($\Delta\varepsilon_{310} = 2.0 \times 10^4$ L·mol$^{-1}$·cm$^{-1}$ for a 15-bp duplex containing three $\mathbf{U^{OH}}$–$Gd^{III}$–$\mathbf{U^{OH}}$ pairs[31]). Source data are provided as a Source Data file.
